# Biochemical Profiling of Urine Metabolome in Premature Infants Based on LC−MS Considering Maternal Influence

**DOI:** 10.3390/nu16030411

**Published:** 2024-01-31

**Authors:** Jeong-Hun Mok, Junhwan Song, Won-Ho Hahn, Seonghyeon Cho, Jong-Moon Park, Jiwon Koh, Ho Kim, Nam Mi Kang

**Affiliations:** 1 Department of Medical Device Management and Research, Samsung Advanced Institute for Health Sciences and Technology (SAIHST), Sungkyunkwan University, Seoul 06355, Republic of Korea; jeonghunmok@naver.com; 2 Department of Pediatrics, Soonchunhyang University Cheonan Hospital, Cheonan 31151, Republic of Korea; joonanii@schmc.ac.kr (J.S.); 99018@schmc.ac.kr (J.K.); c78141@schmc.ac.kr (H.K.); 3 Department of Pediatrics, Soonchunhyang University Seoul Hospital, Seoul 04401, Republic of Korea; dr.hahn.md@gmail.com; 4 Basil Biotech, Incheon 22002, Republic of Korea; shcho@basilbiotech.com (S.C.); basil@basilbiotech.com (J.-M.P.); 5 Department of Nursing, Research Institute for Biomedical & Health Science, Konkuk University, Chungju-si 27478, Republic of Korea

**Keywords:** metabolomics, premature infant, maternal nutrition, LC−MS, human milk, neurotransmitter, amino acid

## Abstract

In this study, Liquid Chromatography–Mass Spectrometry (LC-MS)-based metabolomics profiling was conducted to elucidate the urinary profiles of premature infants during early and late postnatal stages. As a result, we discovered significant excretion of maternal drugs in early−stage infants and identified crucial metabolites like hormones and amino acids. These findings shed light on the maternal impact on neonatal metabolism and underscore the beneficial effects of breastfeeding on the metabolism of essential amino acids in infants. This research not only enhances our understanding of maternal–infant nutritional interactions and their long−term implications for preterm infants but also offers critical insights into the biochemical characteristics and physiological mechanisms of preterm infants, laying a groundwork for future clinical studies focused on neonatal development and health.

## 1. Introduction

The nutrition of premature infants is important for health, optimal growth, and development. The maternal nutritional status, as the primary source of nutrition, significantly influences the nutritional status of premature infants. Poor maternal nutrition can lead to negative birth outcomes, such as low birth weight, and has long−term postnatal effects [1]. Consequently, previous research has focused on maternal–infant substance transfer, including aspects of breastfeeding, obesity, and environmental factors [2,3,4]. Representatively, breastfeeding, as a primary method of nutrition, has been extensively studied for its impact on maternal diet, infant microbiota, and disease or allergy prevention [5,6]. The ongoing focus on mother–infant interaction research underscores its significance, especially for preterm infants with underdeveloped organs. However, previous studies on related biomolecules have often focused on key nutrients such as ATP, glucose, long−chain fatty acids, and amino acids [7,8,9,10,11]. This has led to a relative deficiency in the comprehensive analysis of postnatal metabolic mechanisms in premature infants. Therefore, our study uses metabolomics profiling of the urine of premature infants to elucidate the influence of maternal health on infant metabolic activity.

A previous study on this concept has identified early postnatal metabolic adaptation and maturation alterations, focusing largely on essential energy metabolic cycles using nuclear magnetic resonance (NMR) [12]. In contrast, our study applies Liquid Chromatography–Mass Spectrometry (LC−MS) analysis and provides novel insights into complex molecular mechanisms in premature infants, including a broader examination of breastfeeding and formula feeding. In particular, LC−MS, known for its higher sensitivity and wider metabolite detection range, has enhanced our in−depth exploration [13]. Many studies have collected samples within two weeks after birth as a baseline for investigating the effects of prematurity, and the first three days after birth are considered an extremely preterm period for the organs of infants including the brain [14,15,16,17]. We analyzed urine samples from the immediate postnatal period (1–3 days after birth) as the Early group and two weeks later (13–16 days after birth) as the Late group. Furthermore, we regrouped the samples into a human milk (HM) group and a formula milk feeding (FM) group to assess maternal influence and metabolic activity.

Through this approach, we have identified significant metabolic mechanisms in the urine of premature infants. This suggests new insights into how maternally transferred metabolites can influence neonatal status. Our non−invasive study contributes to a multifaceted evaluation of factors affecting premature infants, leading to a deeper understanding of their health and developmental processes.

## 2. Materials and Methods

### 2.1. Materials

High−performance liquid chromatography (HPLC)−grade water, acetonitrile (ACN), and methanol (MeOH) were purchased from JT Baker (Philipsburg, NJ, USA). Formic acid (FA) was purchased from Sigma−Aldrich (St. Louis, MO, USA). 

### 2.2. Methods

#### 2.2.1. Sample Preparation

Urine samples of 10 mL were collected from premature infants aged 1–3 days (Early group, *n* = 22) and 13–16 days (Late group, *n* = 12, serving as the control) and were subsequently stored at −20 °C. We also recorded whether the infants were fed breast milk (*n* = 14) or formula milk (*n* = 20). The HM group included infants who received 100% human milk and a mixed diet of breast and formula milk, with at least two−thirds breast milk, while the formula milk group consisted exclusively of infants fed 100% formula milk. The urine samples were thawed on ice and mixed with a four times larger volume of chilled MeOH. These mixtures were vortexed for 1 min, centrifuged gently, and then incubated overnight. After incubation, mixtures were centrifuged at 14,000× *g* for 10 min. The supernatants were then transferred to new tubes and dried. Finally, the samples were resuspended in 0.1% FA and prepared for LC−MS analysis.

#### 2.2.2. LC−MS

Chromatographic separation of the samples was performed using an Agilent ZORBAX Eclipse Plus C18 Rapid Resolution High−Definition column (2.1 × 50 mm, 1.8 µm particles) on a Vanquish UHPLC system (Thermo Fisher Scientific, Waltham, MA, USA), equipped with a Q−Exactive Hybrid Quadrupole−Orbitrap MS (Thermo Fisher Scientific, Waltham, MA, USA). The mobile phases consisted of 0.1% FA in water (solvent A) and 0.1% FA in 80% ACN (solvent B), with a flow rate of 200 μL/min. The total gradient time was set at 30 min: 2.5% B for 0–2 min; 2.5–12% B for 2–11 min; 12–28% B for 11–15 min; 28–60% B for 15–22 min; 60–96% B for 22–22.1 min; 96% B for 22.1–24 min; 96–2.5% B for 24–24.1 min; and finally, 2.5% B for 24.1–30 min. Mass spectrometry was conducted in positive electrospray ionization mode, equipped with a Heated Electrospray Ionization Probe, with the resolutions for full−MS and MS/MS scans set at 70,000 and 17,500 (at 400 *m*/*z*), respectively. The scanning range was 100–1000 *m*/*z*, with an automatic gain control (AGC) target of 1 × 10⁶, a maximum IT of 100 ms, and a normalized collision energy (NCE) for dd−MS2 of 30%. For data analysis, Compound Discoverer 3.3.2.31 (Thermo Fisher Scientific, Waltham, MA, USA) was used; this workflow for untargeted metabolomics facilitated retention time alignment and compound identification. MzCloud was employed to annotate compounds at the MS/MS level. The ChemSpider, Human Metabolome Database (HMDB), and Kyoto Encyclopaedia of Genes and Genomes (KEGG) databases were utilized to annotate features based on exact mass, using the internal database of Compound Discoverer. Chemical background noise was eliminated using a blank file.

## 3. Results

We analyzed the metabolism of premature infants in early postnatal development to identify significant molecular mechanisms and enhance our understanding of the biological relationship between mothers and infants. In this study, a total of 34 urine samples were collected from premature infants, divided into two groups: an Early group consisting of infants within 1 to 3 days postnatal and a Late group consisting of infants within 13 to 16 days postnatal. We analyzed the data using LC−MS and applied the Metabolomics Society’s Metabolomics Standards Initiative annotation for standardization [18]. The filtration was performed at Level 2, involving exact mass matching (10 ppm) and a fragmentation score over 80 in the mzCloud database. As a result, 316 metabolites were identified, and 284 metabolites were used in the final analysis, applying a data filtering process, which included using an interquartile range variance filter to exclude the least informative 10% of variables (Table S1).

### 3.1. Multivariate Analysis

**Figure 1:**
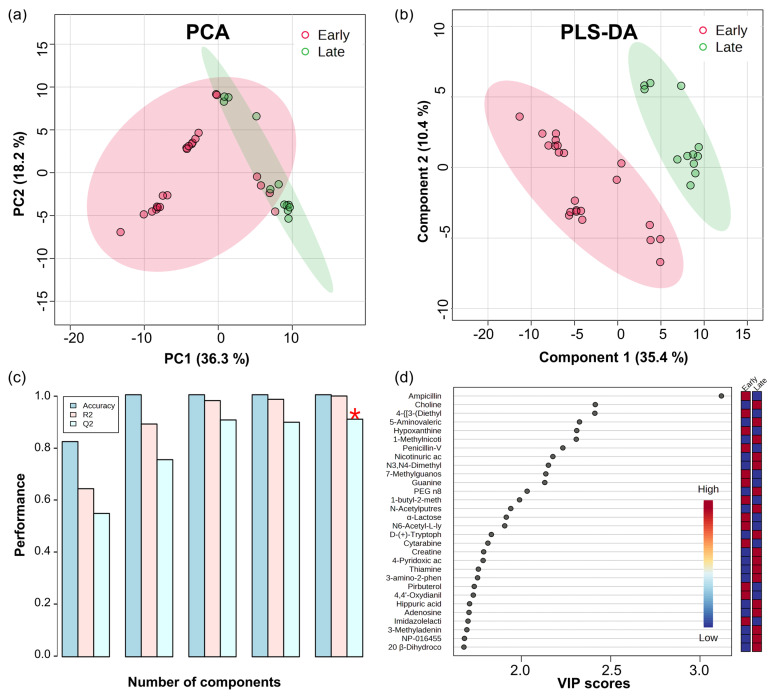
(**a**) PCA plot; (**b**) PLS−DA plot for Early group (red; *n* = 22) and Late group (green; *n* = 12); (**c**) Cross−validation of PLS−DA; (**d**) The top 30 VIP scores. The marker of (*) in (**c**) represents the highest value in the performance measure.

**Figure 2:**
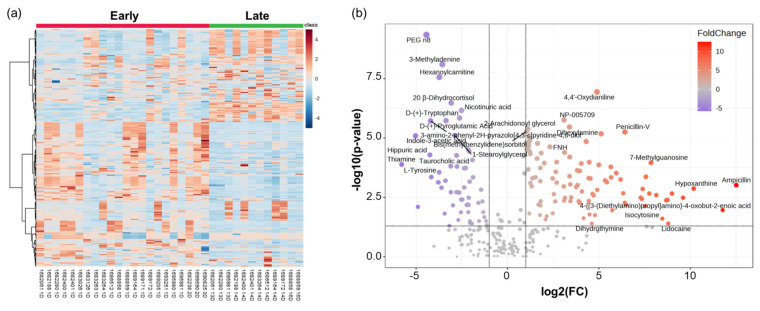
(**a**) Heatmap showing the quantitative values of each compound; (**b**) Volcano plot displaying DEMs (fold change (FC) = Early (*n* = 22)/late (*n* = 12), *p*−value < 0.05, |log2(FC)| > 1).

Initially, a multivariate data matrix was simplified using Principal Component Analysis (PCA) to visualize similarities and differences between the two groups (Figure 1a). The PCA revealed that PC1 and PC2 accounted for 36.3% and 18.2% of the variance, respectively, distinguishing the groups overall, but some overlap in patterns was observed in certain samples. Subsequently, Partial Least Squares Discriminant Analysis (PLS-DA) indicated distinct pattern separation between the Early and Late groups, with the first two components explaining 35.4% and 10.4% of the variance, respectively (Figure 1b). In cross−validation, the model with five components achieved an accuracy of 1.0, an R² of 0.995, and a Q² of 0.907, confirming its high efficacy in differentiating the groups (Figure 1c). Notably, small−scale clustering patterns in each group were consistently observed in both the PCA and PLS−DA results. To identify the metabolites driving these patterns, the top 30 substances with a Variable Importance in Projection (VIP) score above 1 were selected (Figure 1d, Table S2). Remarkably, ampicillin was distinguished by a significantly higher VIP score compared to other metabolites, emerging as a key differentiator between the groups. Moreover, other drugs such as penicillin−V and pirbuterol, as well as common urinary metabolites like α−lactose and creatine, were also identified in Figure 1d. Then, metabolites associated with purine and pyrimidine metabolism, such as hypoxanthine, guanine, thiamine, and adenosine, were identified as significant. The multivariate analysis patterns were consistently reflected in a heatmap displaying the quantitative values of each compound (Figure 2a). The clear separation between the two groups and the small−scale clusters observed in the PCA and PLS−DA results were further clarified by visualizing the results in the heatmap. Specifically, in the Early group, certain metabolites showed quantitative values that indicated overlapping patterns between the groups, reconfirming the presence of overlaps and specific small−scale clusters. Therefore, additional analysis was performed to identify specific metabolites.

### 3.2. Differential Analysis

After observing the differences in patterns between groups, differentially expressed metabolites (DEMs) were identified to specify the significant molecular mechanisms. As a result, 100 upregulated and 56 downregulated metabolites were identified in the Early group and visualized in a volcano plot (Figure 2b). Representatively, drug metabolites including ampicillin, penicillin−V, amoxicillin, and lidocaine were upregulated in the Early group. Furthermore, purine and pyrimidine metabolism-related metabolites (hypoxanthine, guanine, thiamine), steroid hormone−related metabolites (pregnenolone and 5α−pregnan−3,20−dione), and neurotransmitter metabolism−related metabolites (taurine, S−adenosylmethionine, and L−pyroglutamic acid) were mainly upregulated in the Early group. All DEMs are listed in Table S3.

### 3.3. Univariate and Multivariate ROC Curve Analysis

**Figure 3:**
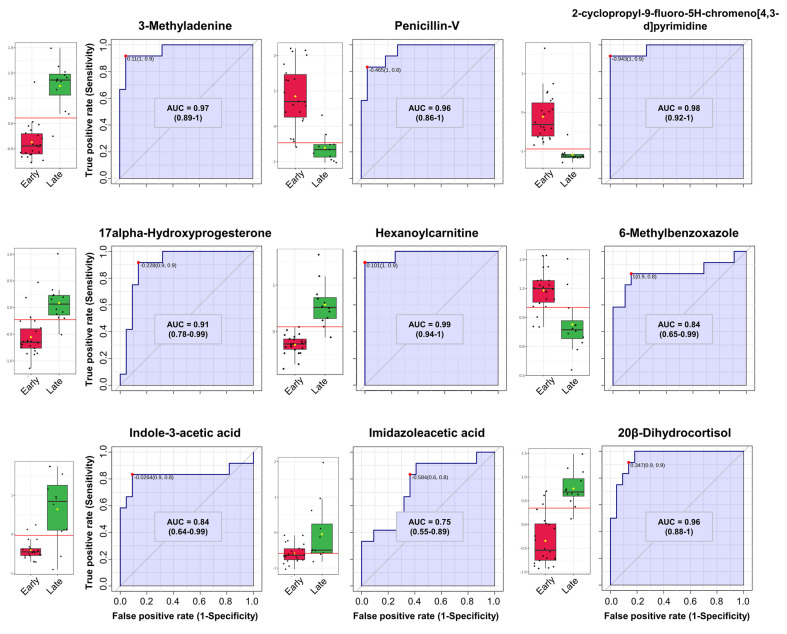
ROC curves with area under the ROC curve (AUC) and confidence interval (CI) values on selected DEMs using the least absolute shrinkage and selection operator (LASSO) feature selection algorithm. Boxplots of relative concentrations for selected DEMs between Early (red) and Late (green) groups. The black dots represent the concentrations of the selected feature from all samples. Horizontal red lines on the boxplot indicate the optimal cutoff. Yellow diamonds represent the mean concentration of each group.

**Figure 4:**
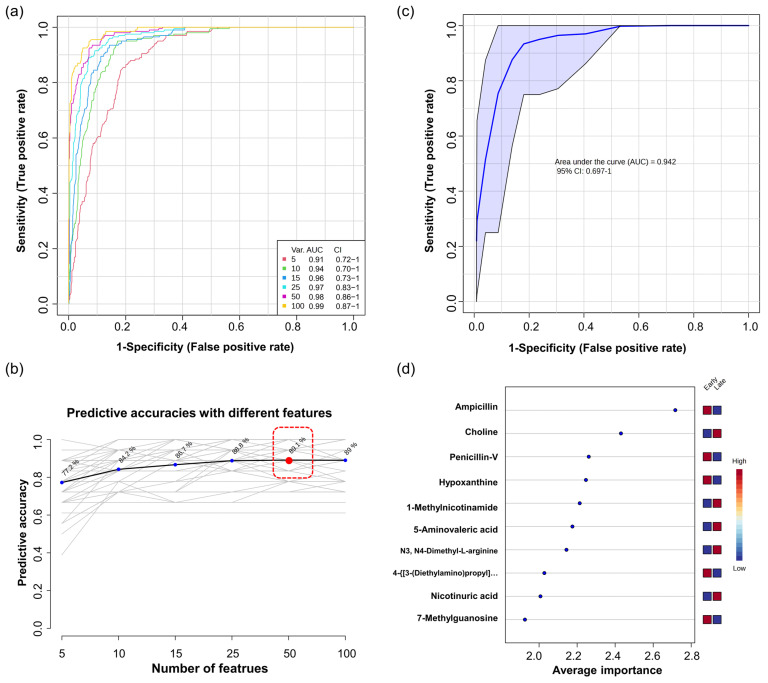
Identification and prediction of key markers between Early group (*n* = 22) and Late group (*n* = 12) using multivariate ROC curve-based exploratory analysis. (**a**) Overview of all ROC curves from six distinct predictive models, highlighting their respective AUC values and CI; (**b**) A chart depicting the predictive performance of each of the six models, with the highest accuracy indicated by a red dot for 50−feature panel of model 5; (**c**) The ROC curve specific to the chosen model 2; (**d**) A list of the top 10 significant metabolites, ranked by their average importance of being selected during cross−validation.

In parallel with other results used to select features in the data, we considered the quantitative regulation of metabolites, VIP scores, and receiver operating characteristic (ROC) curve analysis results. First, we elaborated and validated the significant DEMs through univariate ROC curve analysis. In order to select variables with high reliability, we applied the least absolute shrinkage and selection operator (LASSO) using the R package “glmnet” to select variables for model establishment (Table S4). This predictive model was computed using 10−fold cross−validation. As a result, we identified predictive models of nine metabolites with a good area under the ROC curve and CI, including penicillin−V (AUC = 0.96, CI = 0.86–1), steroid hormones such as 20β−dihydrocortisol (AUC = 0.96, CI = 0.83–1), and 17 α−hydroxyprogesterone (AUC = 0.91, CI = 0.78–0.99) (Figure 3, Table S5). Considering the complexity of the profiling results, driven by interactions among multiple metabolites as variables, an additional predictive model was constructed using multivariate ROC curve analysis to supplement a deeper understanding (Table S6). Multivariate ROC curve analysis was conducted based on Monte Carlo Cross−Validation. The classification method and the feature-ranking mechanism were performed using the PLS−DA algorithm. The ROC curves were generated for models with different numbers of features (5, 10, 15, 25, 50, 100), displaying plots, AUC values, and Cis. The AUC scores ranged from 0.905 to 0.986 (Figure 4a). In predictive accuracy, the 50−feature panel of model 5 achieved the highest accuracy, as shown in Figure 4b. However, to avoid overfitting, we selected the 10−feature panel of model 2. This decision was based on the AUC values exceeding 0.9 for all ROC curves and similar accuracy predictions for more than 10 features. The AUC of model 2 was 0.94 with a 95% CI of 0.687–1, visualized in Figure 4c. From this predictive model, the most significant 10 markers were classified based on average importance (Figure 4d). Consistently, ampicillin, choline, and penicillin−V were significantly reaffirmed and validated in this model as significant metabolites, coordinating with other analytical results.

## 4. Discussion

In this study, we analyzed the differences in urinary metabolites between early− and late−stage preterm infants, examining the relationship with maternal transfer of metabolites. Furthermore, we restructured the sample groups to screen preterm infant urine metabolites from various perspectives, focusing on the effects of breastfeeding as a primary mechanism of substance transfer. The analysis revealed that drugs derived from mothers were most distinctly detected in the urine of early−stage preterm infants. Subsequently, significant metabolites related to physiological mechanisms, such as steroid hormones, amino acids, and nucleic acids, were identified. These findings demonstrate that a variety of metabolites are definitively transferred from the mother to the infant post−birth, with some exogenous substances circulating at high concentrations in the infant’s body and being substantially excreted between 1 and 3 days post−birth. Furthermore, as a result of the analysis regrouping the sample into an HM group and an FM group, upregulation of some essential amino acids and related metabolites was identified in the urine of the HM group. This underscores the positive impact of breastfeeding on essential amino acid metabolism. This study provides a non−invasive, fundamental approach to understanding the biochemical characteristics in preterm infants, underscoring that the identified alterations in metabolites serve as indirect markers for specific molecular mechanisms. These results are expected to offer novel insights into the multidimensional understanding of the physiological mechanisms in preterm infants and the factors influencing their development and health.

### 4.1. Investigating the Diverse Physiological Mechanisms and Drug Metabolism in Preterm Infant Urinary Metabolites Using Multivariate Analysis

Initially, this study determined that the metabolite levels in the Early group indicated considerable variability. This substantial deviation in quantitative values is primarily attributed to the physiological immaturity in preterm infants, manifesting in underdeveloped respiratory [19] and hepatic functions [20,21], among other systems. Reflecting these differences, multivariate analysis results demonstrated specific patterns influenced by multifaceted quantitative values. The PCA plot, while showing some overlap between the Early and Late groups, indicated a high similarity within some samples of the same group, showing a distinction between the two groups overall. This pattern suggests that the variability in the Early group is driven by biases from certain variables. Subsequently, the PLS−DA plot emphasized distinctions between the two groups, though minor sub−clusters within each group were observed. These results imply a significant role of certain variables over time post−birth, underscoring the necessity for further analysis and identification of these variables. A similar pattern was also observed in the heatmap analysis. The formation of small−scale clusters within groups, particularly in the Early group, reaffirmed the hypothesis of considerable variation in the quantitative values of specific variables. This finding highlights the complexity of physiological processes in preterm infants and the need for comprehensive multi−variable analysis to better understand these mechanisms. This study aimed to identify key variables contributing to the multidimensional characteristics of the metabolome in our samples. Our approach involved constructing various predictive models and conducting quantitative comparative analyses. Initially, the PLS−DA model highlighted ampicillin as the most significant variable based on its high VIP score and average importance in multivariate ROC curve analysis. Notably, ampicillin has been extensively reported in various studies, including its use in infant fever [22], pharmacokinetics [23,24], and preterm infants [25,26]. Additionally, penicillin−V was also identified as a significant metabolite, exhibiting a high VIP score and average importance. While penicillin antibiotics are known for their safety and low toxicity in neonates, the severity of the use of empirical antibiotics in preterm infants has also been reported [27,28]. The detection of these drugs in high concentrations in urine and systemic circulation suggests a significant association between material transfer between preterm infants and mothers. Particularly, these findings also suggest the influence of external factors such as maternal nutritional status and treatment methods on the metabolism of preterm infants. However, future studies must recognize the potential impact of these substances as significant confounding factors in experiments. This underscores the importance of employing refined methodologies to comprehend intricate interactions in neonatal metabolism.

### 4.2. Identification of Differential Metabolites in Preterm Infants’ Urine over Time via Differential Expression Analysis

Subsequently, we identified DEMs in preterm infants’ urine to investigate by comparing quantitative alterations post−birth. To select significant DEMs, we considered VIP score values and results from univariate and multivariate ROC curve analyses. The compound−related functionally significant DEMs were investigated as a priority. As a result of cross−validating various analysis methods with high reliability, we confirmed specific physiological mechanisms post−birth, particularly in drugs, hormones, nucleic acids, and amino acid metabolism. Initially, in the Early group, ampicillin showed the highest log2(FC), identified as the most statistically significant variable in multivariate analyses. Different drugs like penicillin−V, amoxicillin, and lidocaine were also upregulated in the Early group. Considering the compromised immunity in pregnant women and the necessity for drugs in childbirth, drugs such as amoxicillin and ampicillin, classified as penicillin−V, along with lidocaine, are known to be safe and have low toxicity for fetuses [29,30]. Our results show that these exogenous metabolites were directly transferred in high concentrations to preterm infants, being excreted in significant amounts after circulating within 1–3 days. However, carefully administering these drugs is necessary, considering the immature hepatic metabolism in preterm infants [31]. Next, significant biological mechanisms involving steroid hormones, purine and pyrimidine metabolism, and amino acid metabolism were also identified. First, DEM−related steroid hormones like 20β−dihydrocortisol, 17α−hydroxyprogesterone, and pregnenolone indicate which specific hormones play important roles in development. Steroid hormones function as essential metabolites in various physiological processes, including development [32,33] and metabolism [34]. Previous research has reported quantitative hormonal alterations in the urine of premature infants during the transition from the types required for intrauterine and independent life [34]. Among these, 20β−dihydrocortisol and 17α−Hydroxyprogesterone were significantly identified in univariate ROC curve analysis and downregulated in the Early group. Research on 20β−dihydrocortisol, a post−metabolic product of cortisol, was reported as a biomarker for Cushing’s syndrome through urine concentration measurements [35]. Additionally, the investigation extends to 17α−Hydroxyprogesterone, a steroid hormone involved in adrenal biosynthesis, transitioning from cholesterol to cortisol [36]. The differential levels of cortisol−related metabolites in preterm infants, depending on the gestational period, underscore their significance in assessing health status [37]. Other upregulated hormone−related substances include pregnenolone as a precursor to steroid hormones [38], 5α−Pregnan−3,20−dione as a neuroactive steroid synthesized from progesterone during fetal development [39], and estriol as a weak estrogen involved in excretion [40]. These differential expressions indirectly provide significant insights into how maternal hormonal states affect metabolic adaptation in preterm infants.

Likewise, some neurotransmitters, similar to hormones, were also differentially expressed in the urine of premature infants. Notably, choline, a precursor to the neurotransmitter acetylcholine and vital for brain differentiation and function, exhibited downregulation in the Early group [41,42]. Choline was identified as a significant metabolite based on its high VIP score and average importance in multivariate ROC curve analysis. Previous research has shown higher concentrations of maternal choline during pregnancy and urinary concentration, with newborns displaying high plasma free choline levels initially, which decrease within the first week [43]. Coupled with this, thiamine was the most significantly downregulated metabolite. Thiamine is a water−soluble vitamin B1 and plays a direct role in the synthesis and release of acetylcholine, with its deficiency linked to reduced acetylcholine levels [44,45]. Considering these, the downregulation of two significant metabolites in the Early group suggests potential negative markers for cognitive and behavioral development in preterm infants. Conversely, other DEMs such as taurine, S−adenosylmethionine, and L−pyroglutamic acid showed significant VIP scores and upregulation trends in the Early group. Taurine, known to function as a neurotransmitter or modulator, has been indicated as a marker for muscle damage from severe exercise [46], while L−pyroglutamic acid is closely related to the major neurotransmitter glutamate and found in high concentrations in urine [47]. Moreover, the increase in neurotransmitter−related metabolite concentrations suggests that these DEMs might be markers of brain diseases or neurodevelopmental disorders for use in urine−based studies of preterm infants. From another perspective, some purine and pyrimidine metabolism− and amino acid−related metabolites were also identified as significant DEMs, participating in fundamental biological mechanisms due to their role in DNA composition, genetic factors, cellular structure, and energy production [48,49,50]. In this study, we observed significant upregulation of hypoxanthine and guanine in the Early group of infants with significant VIP scores and average importance. Hypoxanthine is a primary breakdown product of ATP, and guanine is a representative nucleic acid of DNA building blocks [51]. According to previous research, high urinary hypoxanthine levels can be related to respiratory disorders and deficiencies in hypoxanthine–guanine phosphoribosyltransferase to acute renal failure in infants [52,53]. Additionally, 7−methylguanosine, the modified purine nucleoside, showed significant VIP scores, associated with ischemic diseases and identified as a biomarker in cancer studies [54,55]. In brief, these upregulations suggest significant metabolic mechanisms related to kidney and respiratory development. Furthermore, amino acids, essential for protein construction and energy in preterm infants, were also notably identified [56]. Among the notable DEMs, acetyl−L−carnitine associated with catabolic and anabolic metabolism in the brain as an endogenous intermediate [57], N−acetyl−L−tyrosine administered for stability and enhanced solubility in premature infants during the first postnatal week [58], and other energy metabolism substances such as hexanoylcarnitine [59], as well as fundamental amino acids such as L−tyrosine and L−phenylalanine, have been identified. Amino acids are multifunctional and actively under investigation from various perspectives. Consequently, imbalanced urinary concentrations of amino acids and associated metabolites are indicative of their influence on maternal conditions and neonatal metabolic stress, encouraging deeper exploration associated with these results.

### 4.3. Efficacy of Human Milk in Premature Infants from a Substance Transfer Perspective and Essential Amino Acids

**Figure 5:**
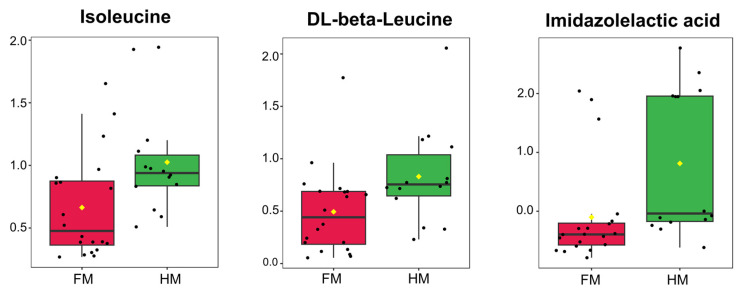
Box–whisker plots of three upregulated metabolites in HM group (*n* = 14) compared to the FM (*n* = 20). Boxplots of relative concentrations for selected DEMs between FM (red) and HM (green) groups. Black dots denote the concentration levels of the chosen feature across all samples. Horizontal red lines mark the optimal cutoff, while the average concentration for each group is symbolized by yellow diamonds.

In this study, we aimed to explore the additional metabolomic profile using preterm infant urine, focusing on another perspective. To investigate this, we regrouped our sample cohorts based on the intake of HM versus FM. Through additional analysis, we revealed that human milk consumption plays a significant role in the supply of essential amino acids. The PCA plot did not show a distinct separation between the HM and FM groups, likely due to the high concentration of specific substances like ampicillin (Figure S1). Nevertheless, among the 12 identified DEMs, isoleucine, imidazolelactic acid, and DL−β−leucine exhibited an intriguing upregulation in the HM group (Figure 5, Table S7). Isoleucine, an essential amino acid involved in the tricarboxylic acid cycle, serves as a fundamental factor in energy metabolism, as well as supplying acetyl−CoA, which is a crucial intermediary in neurotransmitter and steroid synthesis [60,61]. The detection of imidazolelactic acid in urine, which is produced through the breakdown of histidine by an alternative pathway in the absence of histidase, has been reported in several studies [62,63]. Histidine is particularly essential in infancy, and underscored in conjunction with L−tyrosine, previously identified as significant. This characteristic arises due to the immature enzymatic systems in newborns, highlighting their critical metabolic role in preterm infants [64,65]. DL−β−leucine is a less abundant β−amino acid compared to its α−analogues but exists in nature both in free-form and peptide−bound states. Although little research has been conducted, previous research for gestational diabetes mellitus using LC−MS has identified it, suggesting its potential specific functions as analogs of leucine, one of the essential amino acids, in preterm infants [66]. In summary, the upregulation of certain essential amino acids in the HM group indicates a significant impact on metabolism immediately after preterm birth. By focusing on breastfeeding influence in terms of direct nutrient delivery, we investigated its impact. This suggests that human milk feeding, compared to formula feeding, might be more effective in delivering these amino acids that are crucial for development. Certainly, further research is required for a full understanding of the biological meaning, but the accumulation of such data may provide insights into the relationship and molecular mechanisms between the mother and preterm infant in future studies.

## 5. Conclusions

In this study, we used metabolomics to analyze differences in urine metabolites between early− and late−stage preterm infants. As a result, drugs derived from mothers were most prominently detected in the urine of early−stage preterm infants, confirming substantial excretion within 1–3 days post−birth. Various key metabolites, including hormones such as pregnenolone and 20β−dihydrocortisol, purine and pyrimidine metabolism-related metabolites like hypoxanthine and guanine, neurotransmitters including choline and L−pyroglutamic acid, and amino acids such as acetyl−L−carnitine and L−tyrosine, were identified through good predictive models and differential expression analysis, demonstrating statistically validated suitability and performance. Our study used LC−MS to analyze urine samples from preterm infants, similar to previous research, but our approach revealed unique physiological mechanisms, demonstrating that LC−MS offers essential insights into the intricate metabolic processes of preterm infants, despite using similar samples and controls. Additionally, this study suggests the potential involvement of substances in preterm infant development and highlights the positive impact of breastfeeding on essential amino acid metabolism. The non−invasive sampling and high sensitivity of this research indirectly indicate the association between mothers and premature infants, providing insights into their biochemical characteristics and physiological mechanisms. Furthermore, the identification of significant metabolites serving as indirect markers for specific molecular mechanisms contributes to the understanding of preterm infant physiology and potential biomarkers for specific clinical studies.

## Data Availability

Data are contained within the article or Supplementary Materials.

## Supplementary Materials

The following supporting information can be downloaded at https://www.mdpi.com/article/10.3390/nu16030411/s1, Figure S1: PCA plot for HM and FM group; Table S1: List of 284 identified metabolites; Table S2: Top 30 VIP score list in PLS−DA model; Table S3: List of 156 identified DEMs in Early/Late group; Table S4: Th selected metabolite list by LASSO algorithm; Table S5: Univariate ROC curve analysis results; Table S6: Multivariate ROC curve analysis results; Table S7: List of 12 identified DEMs in HM/FM group.
