# Supplementary figures and images for: Biochemical Profiling of Urine Metabolome in Premature Infants Based on LC−MS Considering Maternal Influence
Source: Nutrients. 2024 Jan 31;16(3):411. doi: 10.3390/nu16030411 (PMC10857068; doi:10.3390/nu16030411)

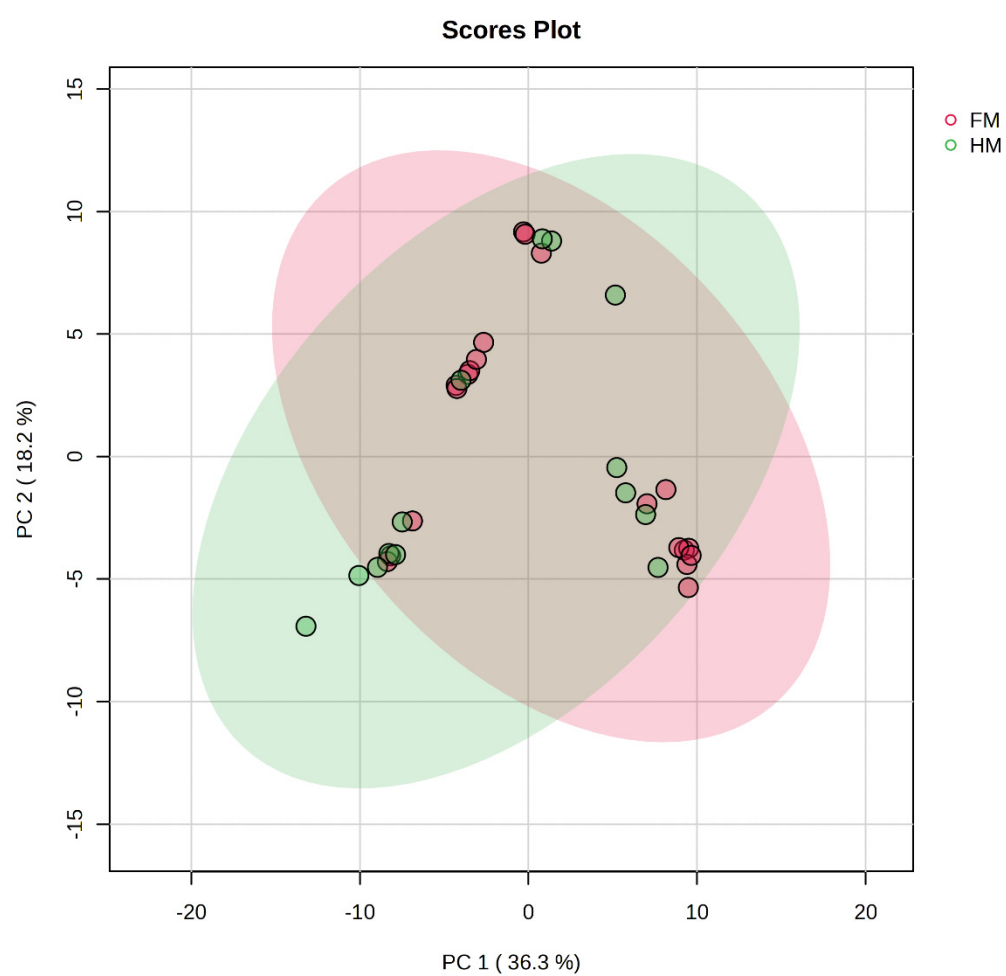

**Figure S1. PCA plot**

Supplement: Supplementary file 1 [file nutrients-16-00411-s001.zip › Supplementary Figure.pdf]
